# Spatiotemporal burden, dietary risk factors, and genetic causality of high body mass index on low back pain: A global, regional, and national analysis based on multi-source databases

**DOI:** 10.1097/MD.0000000000051130

**Published:** 2026-10-23

**Authors:** Houzhen Zhang, Dandan Li

**Affiliations:** a Department of Tuina, Xinjiang Uygur Autonomous Region Academy of Traditional Chinese Medicine, Urumqi, Xinjiang Uygur Autonomous Region, China; b Department of Tuina, The Fourth Clinical College of Xinjiang Medical University, Urumqi, Xinjiang Uygur Autonomous Region, China; c Department of Tuina, The Fourth Affiliated Hospital of Xinjiang Medical University, Urumqi, Xinjiang Uygur Autonomous Region, China.

**Keywords:** exponential smoothing, Global Burden of Disease, Global Dietary Database, high body mass index, low back pain, Mendelian randomization

## Abstract

Low back pain (LBP) is a leading cause of disability worldwide, and high body mass index (BMI) is a modifiable contributor to its burden. We assessed the spatiotemporal burden, dietary context, and genetic evidence linking BMI with LBP. Annual years lived with disability (YLDs) and disability-adjusted life years (DALYs) attributable to high BMI were obtained from the Global Burden of Disease Study 2021 for 1990 to 2021. Trends were evaluated globally, in China, across 6 World Health Organization regions, and across 5 Socio-demographic Index (SDI) quintiles. Estimated annual percentage changes (EAPCs), SDI correlations, and exploratory exponential-smoothing forecasts were calculated. Dietary estimates for 1990 to 2018 were obtained from the Global Dietary Database (GDD). Two-sample Mendelian randomization (MR) was used to evaluate the effect of genetically predicted BMI on LBP. From 1990 to 2021, global DALYs attributable to high BMI increased from 3.09 million to 8.36 million, while the age-standardized DALY rate rose from 70.22 to 97.66 per 1,00,000 population (EAPC 1.14, 95% CI: 1.11–1.17). In China, DALYs increased from 2,45,215.63 to 10,62,017.57 and the rate from 24.34 to 52.70 per 1,00,000 (EAPC 2.89, 95% CI: 2.70–3.09). The global trend was steeper in males than in females (*P* < .001), whereas the difference between Chinese males and females was not statistically significant (*P* = .266). Exploratory forecasts suggested a continued increase in absolute global burden. MR supported a positive association between genetically predicted BMI and LBP (inverse-variance weighted OR 1.007, 95% CI: 1.005–1.009; *P* = 6.40 × 10^–13^). GDD estimates showed higher refined-grain intake in East Asia and higher sugar-sweetened beverage intake in the Americas. High BMI contributes to the growing burden of LBP, but the dietary analyses are ecological and should be interpreted as context rather than evidence of mediation. Weight management and region-specific dietary measures may help reduce this burden.

## 1. Introduction

Low back pain (LBP) is a globally pervasive musculoskeletal disorder, clinically defined as pain localized between the lower margin of the 12th rib and the gluteal folds, with or without referred leg pain.^[1,2]^ For decades, LBP has not only been the primary driver of functional limitation and diminished quality of life but has also remained the single largest contributor to global years lived with disability (YLD). According to recent data from the Global Burden of Disease (GBD) study, the health loss attributed to LBP has surpassed that of major chronic conditions such as diabetes and chronic obstructive pulmonary disease, establishing it as a critical global public health priority.^[3-5]^ While traditional biomechanical models have historically attributed LBP to occupational hazards, structural spinal abnormalities, or acute trauma, emerging epidemiological evidence suggests that the global obesity epidemic and associated metabolic factors – specifically high body mass index (BMI) – are reshaping the epidemiological landscape of LBP.^[6,7]^

Despite its importance, several knowledge gaps remain. Most GBD-based studies have described the overall burden of LBP rather than the burden specifically attributable to high BMI across sex, geography, and development level.^[8,9]^ Observational associations between obesity and back pain may also be affected by socioeconomic position, physical activity, and reverse causation. In addition, the dietary context of high BMI differs across regions, including high refined-grain intake in parts of East Asia and high sugar-sweetened beverage (SSB) intake in the Americas. Large-scale evidence is therefore needed to characterize the attributable burden while separating descriptive dietary patterns from causal inference. The present study integrates GBD 2021 estimates, GDD data, and 2-sample Mendelian randomization (MR) to describe temporal and geographic patterns, explore future burden, and assess genetic evidence for an effect of BMI on LBP. This combination provides a population-level basis for more targeted prevention while recognizing the distinct inferential limits of each data source.

## 2. Materials and methods

### 2.1. Study design, data sources, study period, and geographic groupings

This ecological secondary analysis used publicly available estimates from the Global Burden of Disease Study 2021 (GBD 2021), coordinated by the Institute for Health Metrics and Evaluation. Annual estimates for 1990 to 2021 were obtained from the GBD Results Tool.^[10]^ The analysis was restricted to the outcomes used in this manuscript: YLDs and disability-adjusted life years (DALYs) attributable to high BMI for LBP, expressed as absolute counts and age-standardized rates per 1,00,000 population. Estimates were examined for the global population, China, 5 GBD-defined Socio-demographic Index (SDI) quintiles (high, high-middle, middle, low-middle, and low SDI), and 6 World Health Organization (WHO) regions (African Region, Region of the Americas, South-East Asia Region, European Region, Eastern Mediterranean Region, and Western Pacific Region).

Dietary estimates were obtained from the GDD for the years available in the submitted analyses (1990–2018), with emphasis on refined grains, whole grains, and SSBs by region, age, and sex. Two-sample MR used summary-level genome-wide association study data for BMI from the Genetic Investigation of Anthropometric Traits consortium and LBP outcome data from large European-ancestry resources, including UK Biobank and FinnGen. All GBD estimates, models, tables, and figures were kept within the GBD 2021 cycle so that historical estimates from different GBD cycles were not mixed. Due to the limitations of the rules for GBD data assistance group members, we can only use GBD2021 and cannot choose GBD2023 as analyzable data.

### 2.2. Definitions and burden measures

LBP was defined according to the GBD cause framework. High BMI was treated as a continuous GBD comparative-risk exposure rather than as a binary clinical diagnosis. For adults aged 20 years or older, the GBD theoretical minimum-risk exposure range is approximately 20 to 25 kg/m^2^; for individuals younger than 20 years, age- and sex-specific International Obesity Task Force criteria are used^.[11,12]^

YLDs quantify nonfatal health loss. DALYs are the sum of YLDs and years of life lost (YLL). Because LBP is modeled as a nonfatal cause in this attribution, YLLs are 0, and the attributable DALY and YLD estimates are numerically identical. Both terms are retained because they are standard GBD outputs, but they are not treated as independent outcomes. Age-standardized DALY and YLD rates were expressed per 1,00,000 population. SDI is a composite indicator based on income per capita, educational attainment, and fertility. GBD counts and rates are reported with 95% uncertainty intervals (UIs), whereas estimated annual percentage changes (EAPCs) are reported with 95% confidence intervals (CIs).

### 2.3. Temporal trend analysis and comparison of EAPCs

Long-term trends in age-standardized rates were summarized using ordinary least-squares log-linear regression: $ln\left(R_{t}\right)=\alpha +\beta_{t}+{\;\varepsilon \;}_{t}$, where *R*_*t*_ is the age-standardized rate in calendar year *t*. The EAPC was calculated as $\;100\times \left[exp\left(\beta_{limit}\right)-1\right]$. The 95% CI for beta was calculated as $\hat{\;}\beta \;\pm t_{0.975,n-2}\times SE(\hat{\;}\beta \;)$; the lower and upper limits were then transformed as EAPC=100×[exp(β)−1]. A trend was classified as increasing when the EAPC estimate and the lower CI limit were greater than 0, decreasing when the estimate and upper CI limit were less than 0, and stable when the CI included 0.

Goodness-of-fit of the log-linear specification was evaluated using *R*^2^ and adjusted *R*^2^, residual-versus-fitted plots, and normal Q–Q plots. First-order temporal autocorrelation was examined with the Durbin–Watson statistic. EAPC was interpreted as an average long-term trend rather than evidence of strictly linear change when diagnostic plots suggested nonlinearity. Prespecified male-female comparisons used $\;z=\frac{\beta_{male}-\beta_{female}}{\sqrt{SE(\beta_{male}{)}^{2}+SE(\beta_{female}{)}^{2}}}$, with a 2-sided *P* value.

### 2.4. Correlation analysis with SDI

Pearson correlation coefficients were calculated for the association of SDI with the age-standardized DALY rate (ASDR) and the absolute YLD count across the included location-year observations. Because DALY and YLD estimates were numerically identical, duplicate correlation panels were not retained. Locally estimated scatterplot smoothing curves with 95% CIs were used to display the shape of the associations. These pooled ecological correlations were interpreted descriptively because observations from the same location over time are not independent, and absolute counts are influenced by population size.

### 2.5. Exploratory forecasting

Future burden was explored using exponential smoothing (ES), a time-series method that assigns progressively greater weight to more recent observations. Models were fitted separately to the 1990 to 2021 series for both sexes, females, and males, and point forecasts for absolute DALY counts and ASDRs were generated for 2022–2050. The model does not incorporate policy changes, migration, or external shocks; accordingly, these projections were treated as exploratory extrapolations rather than mechanistic forecasts.

### 2.6. Mendelian randomization analysis

Two-sample MR was performed to evaluate the effect of genetically predicted BMI on LBP. The 3 core assumptions were relevance, independence from confounders, and exclusion restriction. BMI-associated single-nucleotide polymorphisms (SNPs) were selected at genome-wide significance (*P* < 5 × 10^–8^) and clumped at *r*^2^ < 0.001 within a 10,000-kb window; SNPs with *F* statistics < 10 were excluded to reduce weak-instrument bias. The primary estimate used inverse-variance weighted (IVW). Weighted median, MR-Egger, weighted mode, and simple mode estimates were used as sensitivity analyses. Cochran’s *Q* assessed heterogeneity, and the MR-Egger intercept assessed directional horizontal pleiotropy. A random-effects IVW model was used when substantial heterogeneity was present. These diagnostics can identify violations but cannot prove that the MR assumptions hold.

### 2.7. Global Dietary Database analysis

GDD estimates were used to describe the geographic, age-specific, sex-specific, and temporal distribution of refined-grain, whole-grain, and SSB intake. These analyses were ecological and descriptive. They provide regional context for obesity prevention but do not establish that the dietary exposures mediate the relationship between BMI and LBP.

### 2.8. Statistical software

Analyses and visualizations were conducted in R version 4.3.2 (R Foundation for Statistical Computing) using relevant packages, including ggplot2 and TwoSampleMR. A 2-sided *P* value < .05 was considered statistically significant.

## 3. Results

### 3.1. The YLDs, DALYs, and EAPC of low back pain attributable to high body mass index: a comparison of the world and China in 1990 and 2021

Table 1 shows that the global burden of LBP attributable to high BMI increased from 1990 to 2021. Global DALYs rose from 30,86,573.08 (95% UI 3,12,559.12 – 64.84,427.06) to 83,63,759.33 (8,40,306.54–1,74,24,821.68), an increase of approximately 171%. The ASDR increased from 70.22 (7.14–146.48) to 97.66 (9.78–204.00) per 1,00,000 population, with an EAPC of 1.14 (95% CI: 1.11–1.17). YLD and DALY estimates were identical because LBP contributed no YLLs in this analysis. Globally, the male EAPC was 1.29 (1.27–1.31), compared with 1.09 (1.05–1.14) in females; the difference in log-linear slopes was statistically significant (*P* < .001). Although females had the higher absolute burden throughout the period, the global trend was steeper in males.

**Table 1:** YLDs, DALYs, and EAPC of low Back pain attributable to high body mass index: a comparison of the world, China in 1990 and 2021.

Location	DALYs		DALYs		1990–2021 EAPC	YLDSs		YLDSs		1990–2021 EAPC
	1900	2021	1900	2021	1990–2021	1900	2021	1900	2021	1990–2021
	Cases (95% UIz	Cases (95% UI)	Rate (95% UI)	Rate (95% UI)		Cases (95% UI)	Cases (95% UI)	Rate (95% UI)	Rate (95% UI)	
Global	30,86,573.08 (3,12,559.12, 64,84,427.06)	83,63,759.33 (8,40,306.54, 1,74,24,821.68)	70.22 (7.14, 146.48)	97.66 (9.78, 204.00)	1.14 (1.11, 1.17)	30,86,573.08 (3,12,559.12, 64,84,427.06)	83,63,759.33 (8,40,306.54, 1,74,24,821.68)	70.22 (7.14, 146.48)	97.66 (9.78, 204.00)	1.14 (1.11, 1.17)
High SDI	12,18,421.82 (1,19,569.00, 25,49,631.15)	24,78,626.07 (2,49,211.24, 50,91,840.29)	118.84 (11.60, 248.80)	161.80 (16.00, 332.59)	1.06 (1.02, 1.09)	12,18,421.82 (1,19,569.00, 25,49,631.15)	24,78,626.07 (2,49,211.24, 50,91,840.29)	118.84 (11.60, 248.80)	161.80 (16.00, 332.59)	1.06 (1.02, 1.09)
High-middle SDI	9,56,976.62 (96,319.02, 20,14,411.50)	20,86,188.17 (2,12,179.44, 42,63,358.45)	91.90 (9.24, 192.00)	115.59 (11.61, 238.00)	0.78 (0.72, 0.84)	9,56,976.62 (96,319.02, 20,14,411.50)	20,86,188.17 (2,12,179.44, 42,63,358.45)	91.90 (9.24, 192.00)	115.59 (11.61, 238.00)	0.78 (0.72, 0.84)
Middle SDI	5,47,306.42 (57,862.32, 11,53,131.65)	22,05,381.99 (2,19,659.90, 46,78,252.07)	41.92 (4.49, 87.56)	79.10 (7.86, 168.24)	2.18 (2.13, 2.22)	5,47,306.42 (57,862.32, 11,53,131.65)	22,05,381.99 (2,19,659.90, 46,78,252.07)	41.92 (4.49, 87.56)	79.10 (7.86, 168.24)	2.18 (2.13, 2.22)
Low-middle SDI	2,76,060.91 (29,215.80, 5,73,856.68)	12,27,064.64 (1,22,732.05, 25,54,600.60)	35.21 (3.78, 72.71)	71.69 (7.24, 148.95)	2.46 (2.41, 2.52)	2,76,060.91 (29,215.80, 5,73,856.68)	12,27,064.64 (1,22,732.05, 25,54,600.60)	35.21 (3.78, 72.71)	71.69 (7.24, 148.95)	2.46 (2.41, 2.52)
Low SDI	82,343.43 (9051.79, 1,65,429.39)	3,56,409.63 (35,002.73, 7,29,956.95)	27.81 (3.09, 55.76)	50.30 (5.07, 103.18)	1.98 (1.92, 2.04)	82,343.43 (9051.79, 1,65,429.39)	3,56,409.63 (35,002.73, 7,29,956.95)	27.81 (3.09, 55.76)	50.30 (5.07, 103.18)	1.98 (1.92, 2.04)
African Region	1,29,992.24 (13,700.96, 2,67,237.18)	5,58,889.95 (55,102.18, 11,70,756.68)	45.65 (4.87, 92.89)	79.72 (8.08, 164.94)	1.79 (1.76, 1.81)	1,29,992.24 (13,700.96, 2,67,237.18)	5,58,889.95 (55,102.18, 11,70,756.68)	45.65 (4.87, 92.89)	79.72 (8.08, 164.94)	1.79 (1.76, 1.81)
Region of the Americas	8,75,210.50 (83,623.38, 18,46,079.37)	21,81,828.60 (2,17,083.75, 44,76,588.21)	135.46 (12.99, 286.08)	180.21 (17.78, 370.23)	0.99 (0.95, 1.03)	8,75,210.50 (83,623.38, 18,46,079.37)	21,81,828.60 (2,17,083.75, 44,76,588.21)	135.46 (12.99, 286.08)	180.21 (17.78, 370.23)	0.99 (0.95, 1.03)
European Region	12,90,175.20 (1,26,897.25, 27,05,328.53)	22,63,930.01 (2,26,370.30, 46,30,727.37)	128.24 (12.56, 269.31)	169.94 (16.70, 351.45)	0.95 (0.93, 0.96)	12,90,175.20 (1,26,897.25, 27,05,328.53)	22,63,930.01 (2,26,370.30, 46,30,727.37)	128.24 (12.56, 269.31)	169.94 (16.70, 351.45)	0.95 (0.93, 0.96)
South-East Asia Region	1,78,381.82 (19,513.65, 3,61,989.50)	9,01,991.76 (86,962.48, 18,45,692.70)	18.94 (2.10, 38.63)	43.68 (4.25, 89.31)	2.92 (2.82, 3.02)	1,78,381.82 (19,513.65, 3,61,989.50)	9,01,991.76 (86,962.48, 18,45,692.70)	18.94 (2.10, 38.63)	43.68 (4.25, 89.31)	2.92 (2.82, 3.02)
Western Pacific Region	4,04,282.15 (47,717.54, 8,34,314.11)	14,67,283.56 (1,52,746.73, 30,55,191.83)	30.07 (3.57, 61.63)	55.98 (5.77, 116.71)	2.24 (2.09, 2.38)	4,04,282.15 (47,717.54, 8,34,314.11)	14,67,283.56 (1,52,746.73, 30,55,191.83)	30.07 (3.57, 61.63)	55.98 (5.77, 116.71)	2.24 (2.09, 2.38)
Eastern Mediterranean Region	1,92,298.85 (18,969.66, 4,09,008.96)	9,35,686.70 (94,900.01, 19,04,765.36)	80.89 (8.12, 171.14)	149.19 (15.18, 300.65)	2.09 (2.06, 2.13)	1,92,298.85 (18,969.66, 4,09,008.96)	9,35,686.70 (94,900.01, 19,04,765.36)	80.89 (8.12, 171.14)	149.19 (15.18, 300.65)	2.09 (2.06, 2.13)
China	2,45,215.63 (29,997.36, 5,00,109.88)	10,62,017.57 (1,09,970.69, 22,25,148.90)	24.34 (2.99, 49.33)	52.70 (5.41, 110.10)	2.89 (2.70, 3.09)	2,45,215.63 (29,997.36, 5,00,109.88)	10,62,017.57 (1,09,970.69, 22,25,148.90)	24.34 (2.99, 49.33)	52.70 (5.41, 110.10)	2.89 (2.70, 3.09)

CI = confidence interval, DALY = disability-adjusted life year, EAPC = estimated annual percentage change, SDI = Socio-demographic Index, UI = uncertainty interval, YLD = years lived with disability.

In China, DALYs increased from 2,45,215.63 (95% UI 29,997.36–5,00,109.88) in 1990 to 10,62,017.57 (1,09,970.69–22,25,148.90) in 2021, corresponding to a 333% increase. The ASDR rose from 24.34 (2.99–49.33) to 52.70 (5.41–110.10) per 1,00,000, and the EAPC was 2.89 (95% CI: 2.70–3.09). Sex-specific EAPCs were 2.78 (2.65–2.91) in males and 2.93 (2.70–3.16) in females; this difference was not statistically significant (*P* = .266). Thus, China experienced a faster increase than the global average, while the data did not support a sex difference in the rate of increase within China (Table 2).

**Table 2:** YLDs, DALYs, and EAPC of low back pain attributable to high body mass index: a comparison of the world, China in 1990 and 2021 (male and female).

Location	YLDs (male)		YLDs (male)		1990–2021 EAPC	YLDs (female)		YLDs (female)		1990–2021 EAPC
	1900	2021	1900	2021	1990–2021	1900	2021	1900	2021	1990–2021
	Cases (95% UI)	Cases (95% UI)	Rate (95% UI)	Rate (95% UI)		Cases (95% UI)	Cases (95% UI)	Rate (95% UI)	Rate (95% UI)	
Global	10,14,232.99 (1,04,682.54, 21,15,704.37)	28,22,385.08 (2,83,740.58, 57,86,405.47)	46.57 (4.84, 96.25)	67.56 (6.78, 138.87)	1.29 (1.27, 1.31)	20,72,340.09 (2,07,876.58, 43,68,722.69)	55,41,374.25 (5,56,565.96, 1,16,36,895.13)	92.01 (9.25, 193.20)	126.29 (12.63, 266.01)	1.09 (1.05, 1.14)
High SDI	4,63,875.01 (45,755.64, 9,75,889.32)	10,03,475.01 (1,01,212.17, 20,50,414.58)	95.48 (9.41, 201.07)	134.88 (13.40, 277.88)	1.19 (1.13, 1.24)	7,54,546.81 (73,813.37, 15,93,850.86)	14,75,151.06 (1,47,999.07, 30,16,875.75)	139.30 (13.51, 294.19)	188.09 (18.71, 384.24)	1.02 (1.00, 1.05)
High-middle SDI	2,84,728.06 (29,221.89, 6,01,696.97)	6,77,038.60 (68,272.90, 14,03,388.71)	57.57 (5.93, 119.89)	79.20 (7.91, 164.69)	1.09 (1.04, 1.13)	6,72,248.56 (67,097.13, 14,08,160.29)	14,09,149.57 (1,43,906.54, 28,69,424.09)	120.96 (12.04, 252.65)	148.93 (15.00, 306.13)	0.70 (0.64, 0.77)
Middle SDI	1,67,249.89 (18,392.11, 3,49,855.98)	6,76,505.05 (67,391.58, 14,25,828.90)	25.12 (2.81, 51.88)	49.50 (4.93, 104.76)	2.32 (2.29, 2.34)	3,80,056.52 (39,470.20, 8,03,844.29)	15,28,876.93 (1,52,268.32, 32,44,438.81)	58.73 (6.17, 123.38)	107.66 (10.69, 228.98)	2.08 (2.02, 2.13)
Low-middle SDI	74,560.72 (8457.60, 1,53,202.66)	3,52,309.62 (35,130.01, 7,45,814.35)	18.77 (2.16, 38.06)	41.96 (4.25, 88.69)	2.76 (2.70, 2.82)	2,01,500.19 (20,758.21, 4,20,454.86)	8,74,755.01 (87,518.11, 18,14,784.82)	52.22 (5.45, 107.58)	100.42 (10.13, 207.43)	2.28 (2.22, 2.33)
Low SDI	21,847.05 (2593.80, 43,735.72)	1,09,481.99 (10,883.56, 2,21,900.29)	14.72 (1.76, 29.09)	31.49 (3.21, 63.91)	2.53 (2.48, 2.58)	60,496.37 (6399.94, 1,23,596.99)	2,46,927.64 (24,106.67, 5,08,524.41)	41.21 (4.41, 84.08)	68.60 (6.85, 141.10)	1.71 (1.65, 1.77)
African Region	34,055.83 (3857.75, 68,925.06)	1,66,418.94 (16,545.61, 3,46,583.70)	24.03 (2.72, 48.50)	50.00 (5.10, 103.25)	2.35 (2.33, 2.37)	95,936.40 (9807.10, 1,96,735.57)	3,92,471.01 (38,556.57, 8,21,250.69)	67.04 (6.92, 136.84)	106.78 (10.78, 220.13)	1.49 (1.46, 1.52)
Region of the Americas	31,1358.01 (30,035.27, 6,65,565.30)	8,22,016.96 (81,592.51, 16,86,050.37)	100.56 (9.75, 214.52)	141.98 (13.99, 292.22)	1.19 (1.11, 1.28)	5,63,852.49 (53,588.11, 11,94,115.53)	13,59,811.64 (1,35,491.24, 28,00,002.65)	166.95 (15.90, 353.57)	215.61 (21.42, 444.11)	0.89 (0.86, 0.92)
European Region	4,12,820.44 (41,105.18, 8,59,613.02)	7,67,984.63 (76,555.62, 15,68,448.92)	91.49 (9.10, 189.83)	125.41 (12.33, 259.59)	1.07 (1.05, 1.08)	8,77,354.76 (85,792.07, 18,45,715.51)	14,95,945.38 (1,49,814.68, 30,71,052.72)	157.80 (15.34, 333.87)	209.50 (20.56, 431.78)	0.95 (0.93, 0.97)
South-East Asia Region	47,512.39 (5414.82, 96,294.47)	2,13,446.07 (21,557.07, 4,39,162.18)	9.77 (1.14, 19.77)	20.78 (2.08, 42.41)	2.68 (2.55, 2.81)	1,30,869.43 (14,098.83, 2,65,733.07)	6,88,545.69 (65,613.87, 14,16,741.70)	28.58 (3.11, 57.93)	66.12 (6.34, 135.69)	2.91 (2.83, 3.00)
Western Pacific Region	1,43,231.43 (17,126.13, 2,95,294.22)	4,91,195.87 (51,208.08, 10,17,035.74)	20.76 (2.51, 42.42)	39.06 (4.03, 81.18)	2.22 (2.13, 2.30)	2,61,050.72 (30,591.40, 5,38,002.01)	9,76,087.69 (1,01,538.65, 20,40,485.54)	39.05 (4.59, 79.99)	72.35 (7.45, 151.45)	2.25 (2.07, 2.43)
Eastern Mediterranean Region	58,973.16 (6134.20, 1,25,010.46)	3,41,989.25 (33,952.96, 7,02,527.06)	47.83 (5.06, 100.82)	104.87 (10.50, 214.86)	2.64 (2.61, 2.68)	1,33,325.68 (12,791.49, 2,82,696.77)	5,93,697.45 (60,947.05, 12,02,709.56)	117.21 (11.43, 246.97)	197.50 (20.28, 394.34)	1.80 (1.77, 1.84)
China	90,422.78 (11,085.62, 1,86,918.73)	3,58,719.17 (36,840.02, 7,45,322.46)	17.12 (2.12, 35.11)	37.45 (3.81, 77.55)	2.78 (2.65, 2.91)	1,54,792.84 (18,911.74, 3,15,730.39)	7,03,298.40 (73,130.67, 14,78,284.51)	31.60 (3.87, 64.09)	67.51 (6.96, 142.32)	2.93 (2.70, 3.16)

CI = confidence interval, DALY = disability-adjusted life year, EAPC = estimated annual percentage change, SDI = Socio-demographic Index, UI = uncertainty interval, YLD = years lived with disability.

### 3.2. The trend of YLDs and DALYs of low back pain attributable to high body mass index

Figures 1 and 2 separate the temporal trends by geographic framework. Figure 1 presents the global, China, and 6 WHO-region series; Figure 2 presents the 5 SDI-quintile series. From 1990 to 2021, absolute YLD/DALY counts and age-standardized rates increased in each WHO region and SDI quintile, although the magnitude and slope differed across locations. All rates are expressed per 1,00,000 population (Table 2).

**Figure 1.**
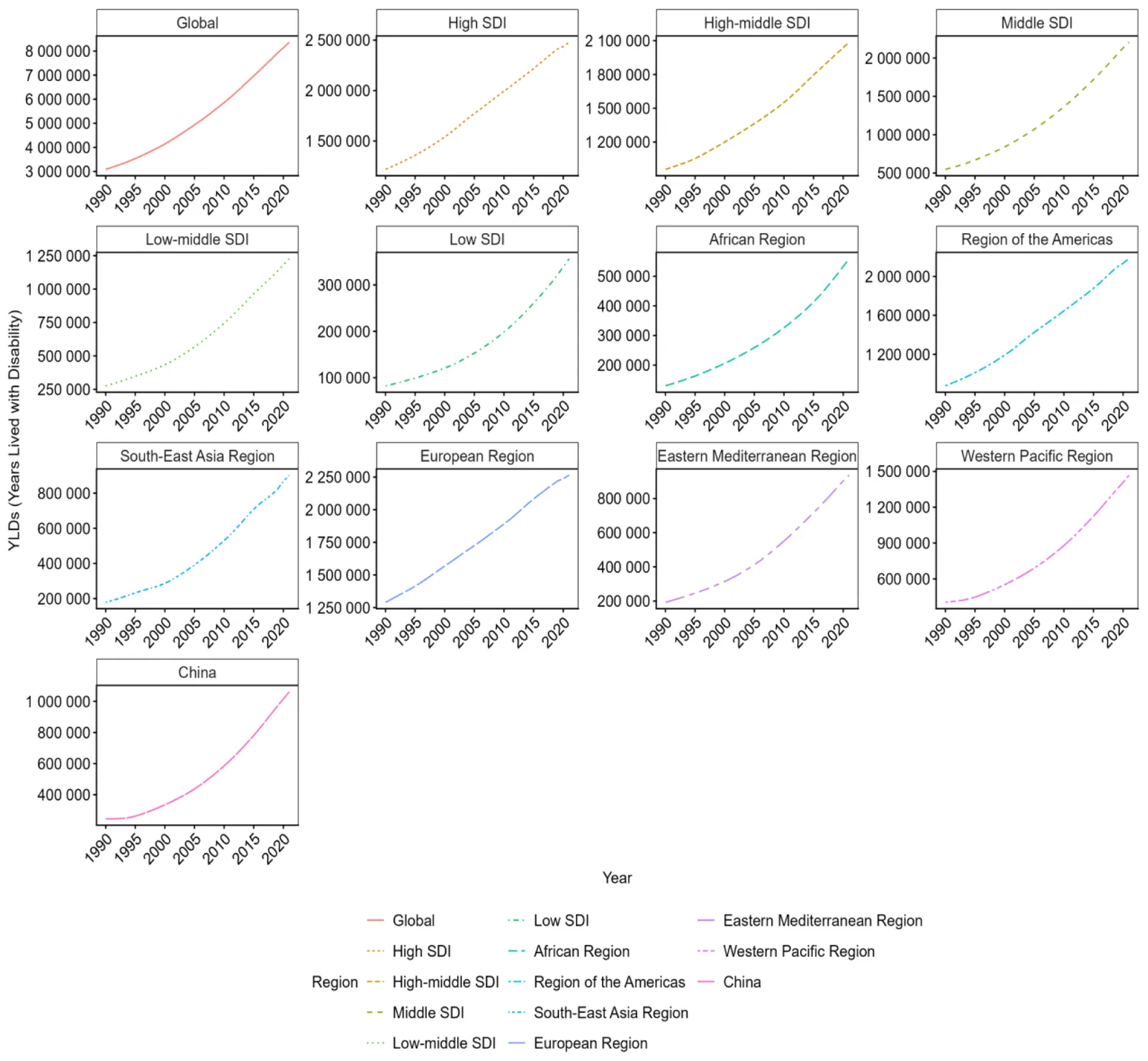
Temporal trends in the burden of low back pain attributable to high body mass index by geographic location, 1990 to 2021. Panel A shows absolute YLD/DALY counts; panel B shows age-standardized YLD/DALY rates per 1,00,000 population. The figure includes the global estimate, China, and the 6 WHO regions. DALY = disability-adjusted life years, WHO = World Health Organization, YLD = years lived with disability.

**Figure 2.**
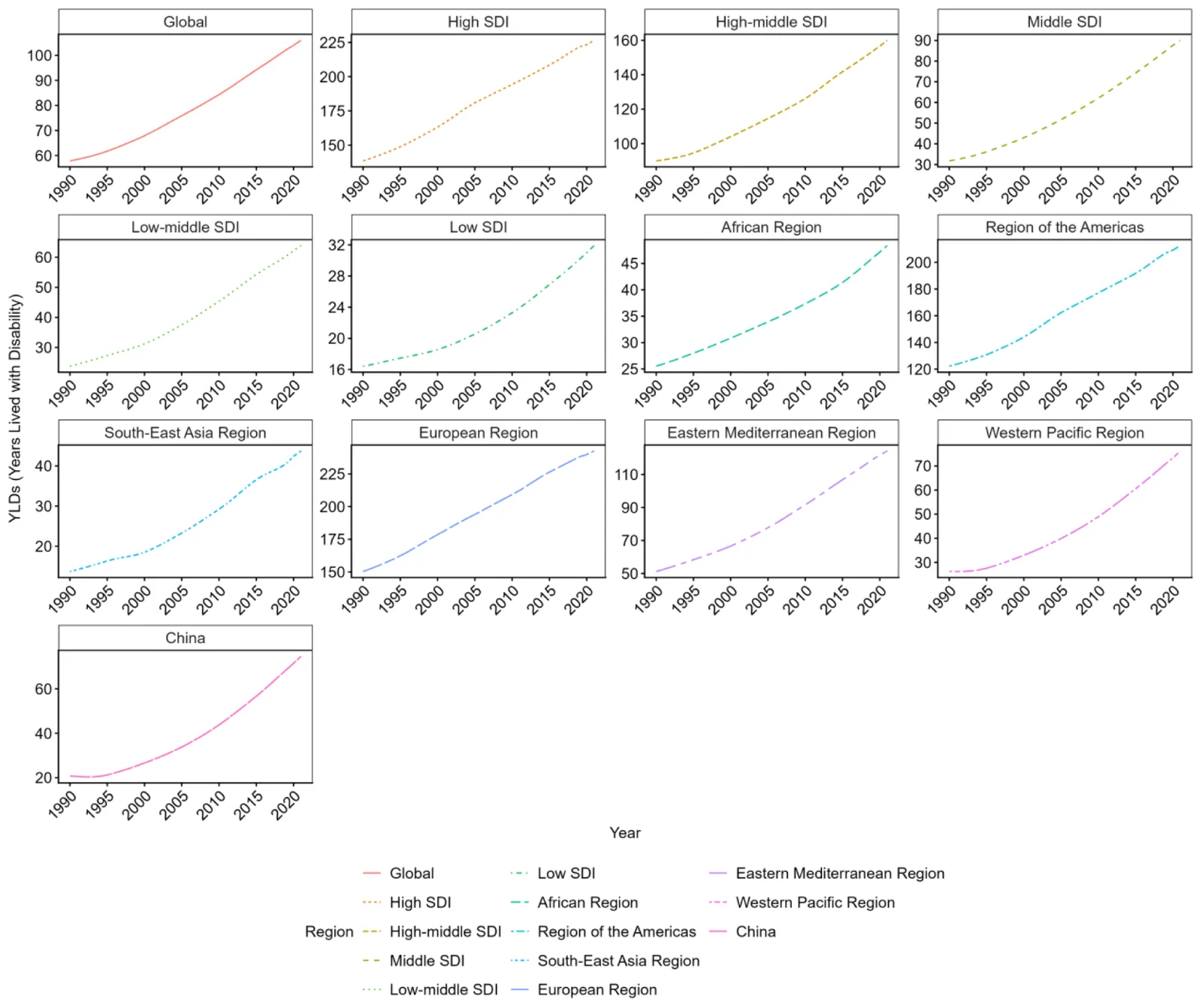
Temporal trends in the burden of low back pain attributable to high body mass index by SDI quintile, 1990 to 2021. Panel A shows absolute YLD/DALY counts; panel B shows age-standardized YLD/DALY rates per 1,00,000 population. DALY = disability-adjusted life year, SDI = Socio-demographic Index, YLD = years lived with disability.

### 3.3. Sex-specific trends in YLDs and DALYs of low back pain attributable to high body mass index

Figures 3 and 4 show sex-specific global trends. Female absolute YLD/DALY counts and age-standardized rates remained higher than male estimates throughout 1990 to 2021. However, the increase in the global age-standardized rate was steeper in males, consistent with the EAPC comparison reported above.

**Figure 3.**
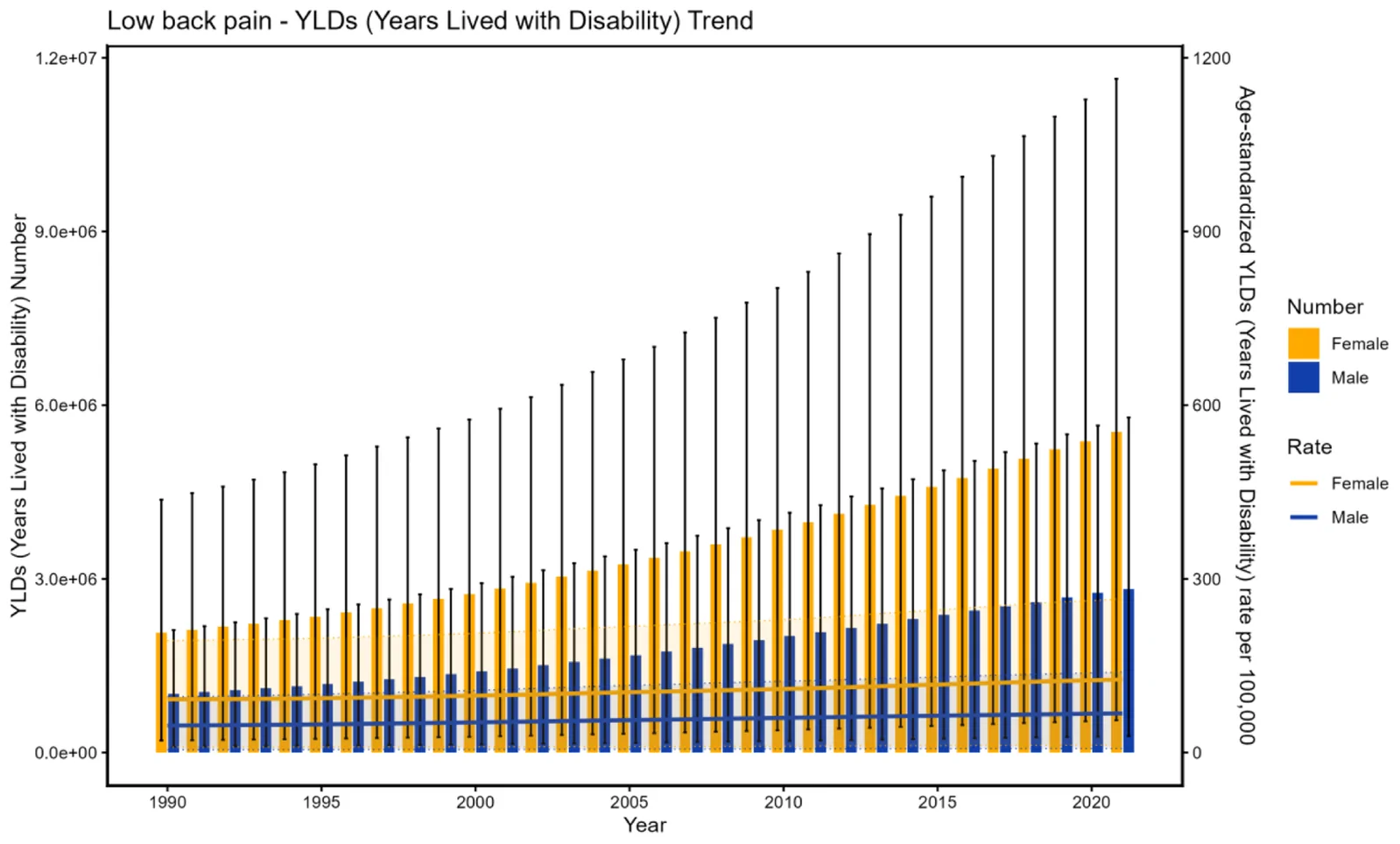
Global sex-specific trends in YLDs attributable to high body mass index, 1990 to 2021. Bars represent absolute numbers (left axis), and lines represent age-standardized rates per 1,00,000 population (right axis). Orange indicates females and blue indicates males. YLD = years lived with disability.

**Figure 4.**
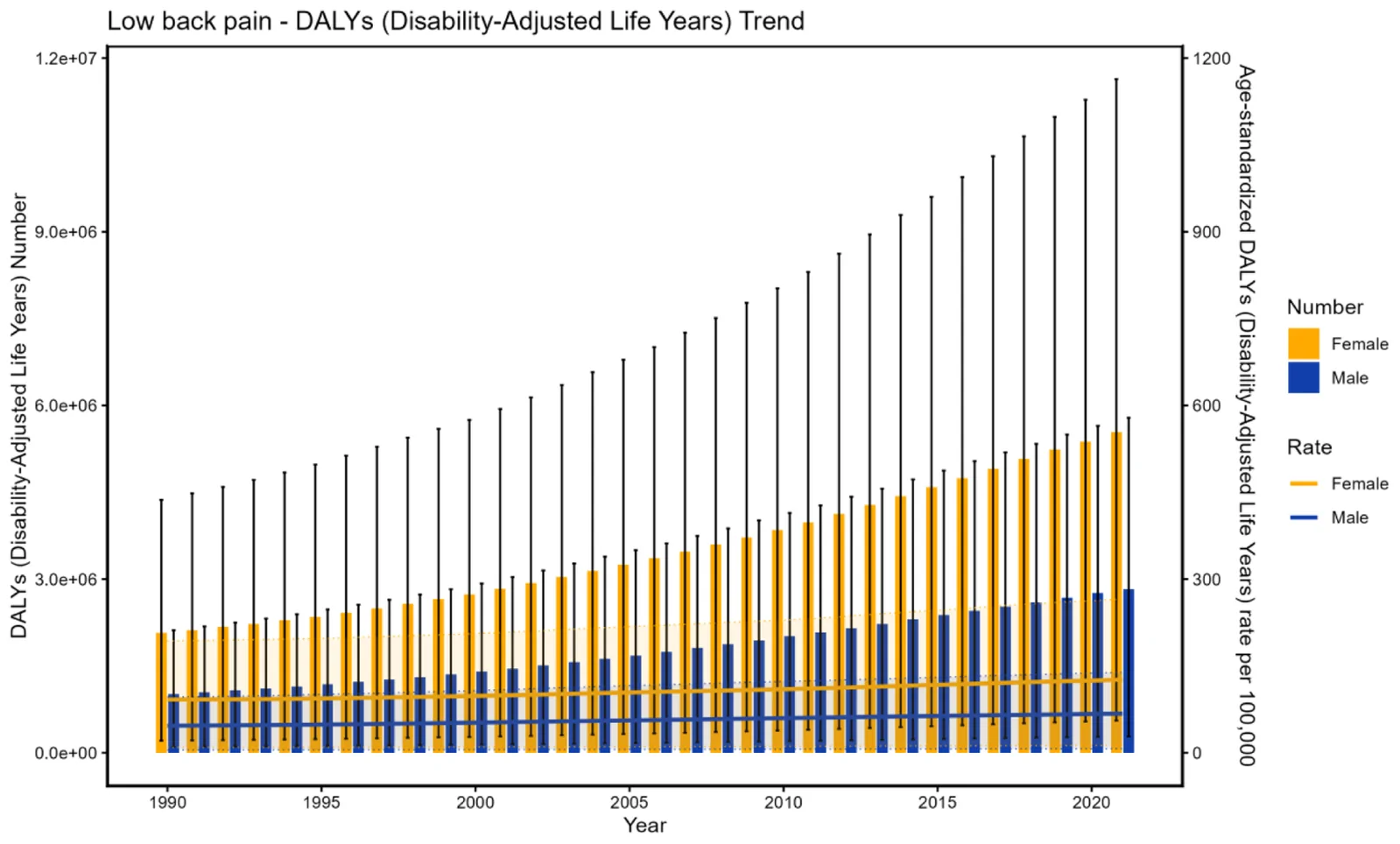
Global sex-specific trends in DALYs attributable to high body mass index, 1990 to 2021. Bars represent absolute numbers (left axis), and lines represent age-standardized rates per 1,00,000 population (right axis). Orange indicates females and blue indicates males. DALY = disability-adjusted life year.

### 3.4. Burden of YLDs and DALYs of low back pain attributable to high body mass index by SDI

Figure 5 shows the ecological association between SDI and the burden attributable to high BMI. SDI was positively correlated with ASDR (*r* = 0.853, *P* < .001) and with absolute YLD count (*r* = 0.824, *P* < .001). The locally estimated scatterplot smoothing curve indicated that the relationship was not strictly linear. Because absolute counts also reflect population size and the observations are repeated within locations, these findings are descriptive and should not be interpreted as a causal effect of development. The redundant DALY/YLD correlation figure in the submitted version was removed.

**Figure 5.**
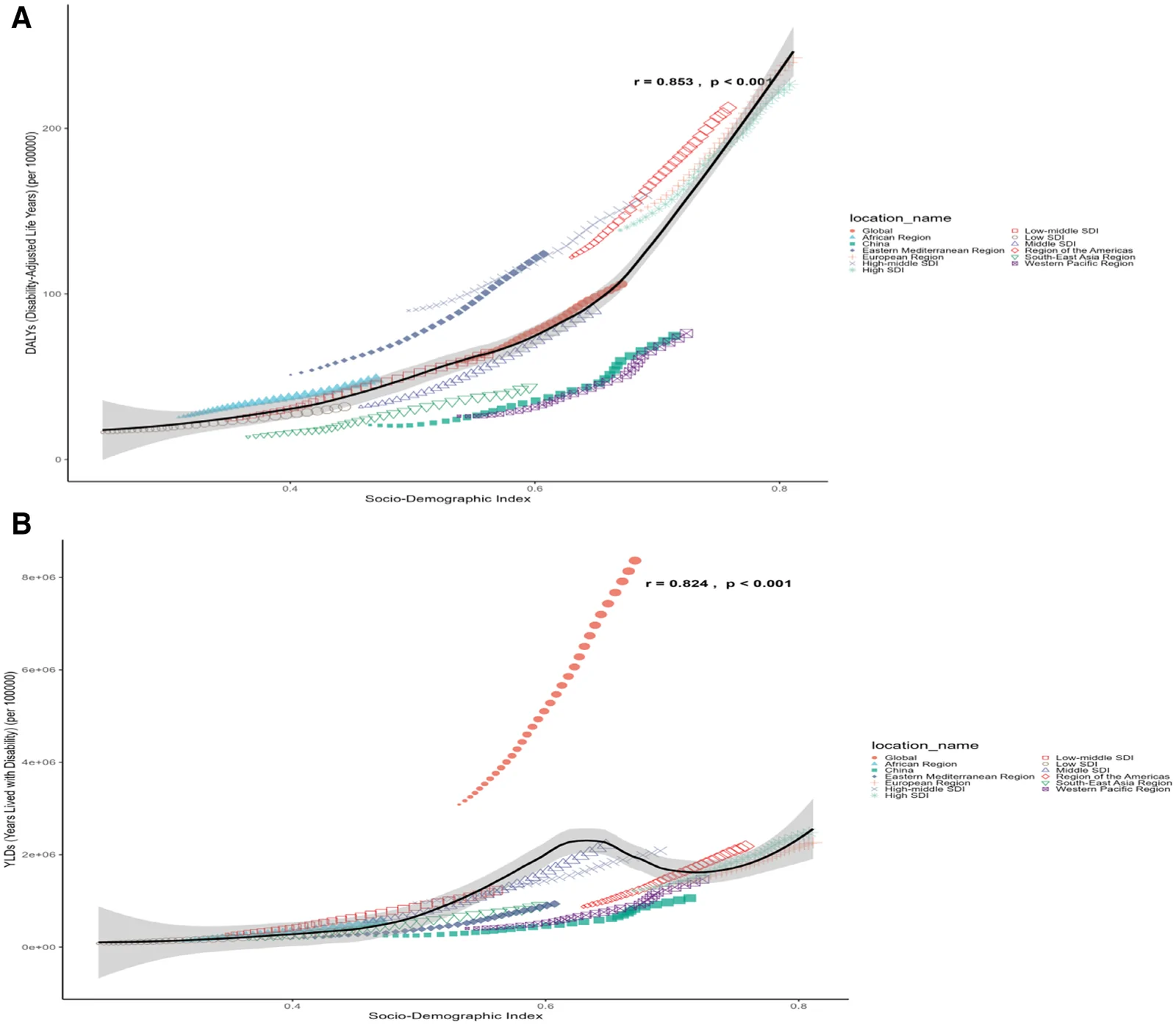
Ecological association between SDI and the burden of low back pain attributable to high body mass index, 1990 to 2021. Panel A shows the age-standardized DALY rate per 1,00,000 population; panel B shows absolute YLD counts. Colored symbols represent location-year observations, and the black LOESS curve is shown with its 95% confidence band. Pearson correlation coefficients and 2-sided *P* values are displayed. DALY = disability-adjusted life year, LOESS = locally estimated scatterplot smoothing; SDI = Socio-demographic Index, YLD = years lived with disability.

### 3.5. Projected disease burden in the world over the next 29 years

As shown in Figure 6, the exploratory ES projections suggested that absolute global DALYs attributable to high BMI would continue to increase through 2050 in both sexes combined, females, and males. The projected female burden remained higher than the male burden. These projections extend the observed 1990 to 2021 trajectories and do not account for future interventions or structural changes.

**Figure 6.**
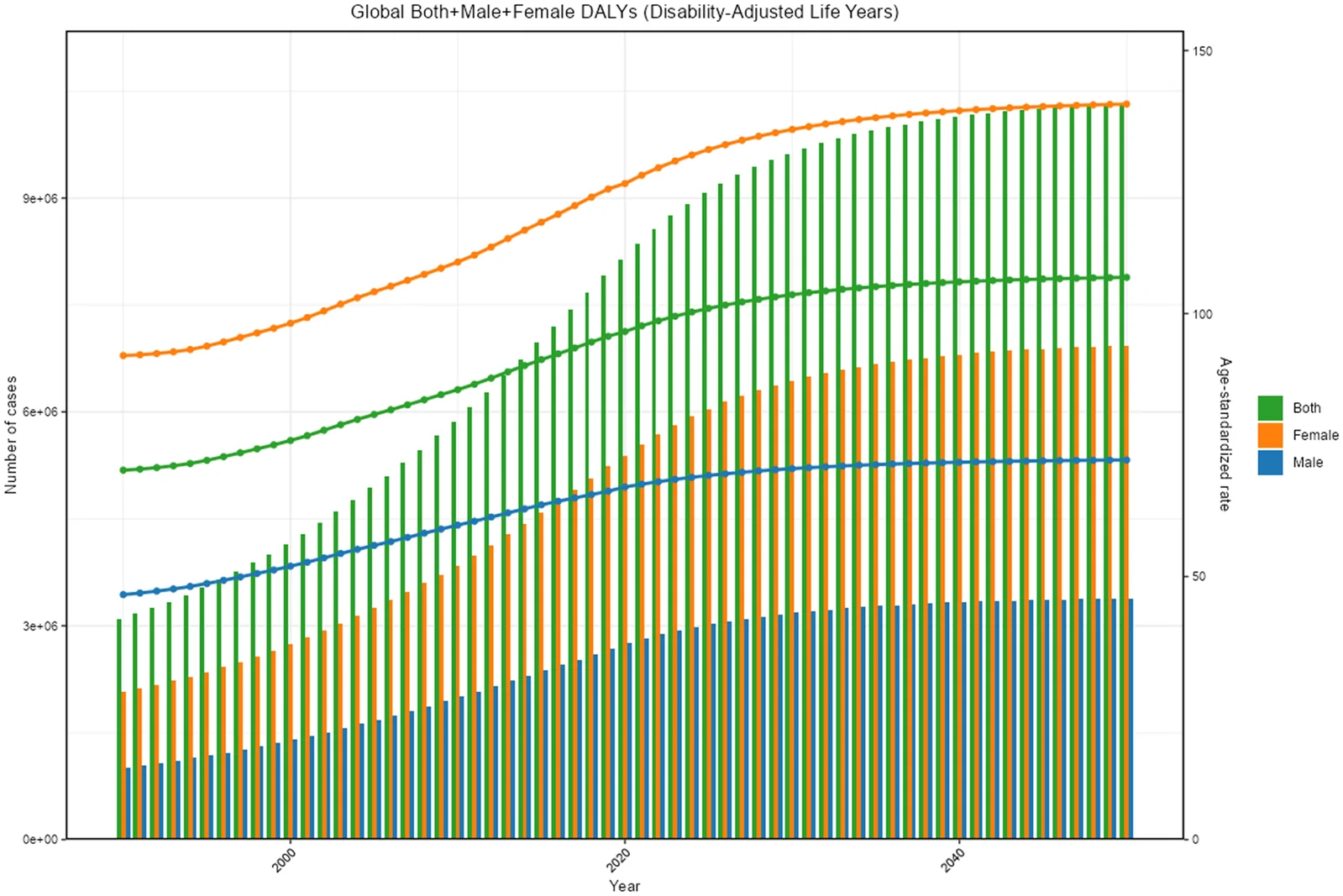
Exploratory exponential-smoothing projections of global DALYs attributable to high body mass index through 2050, by sex. Bars show absolute DALY counts (left axis), and lines show age-standardized DALY rates per 1,00,000 population (right axis). Green indicates both sexes combined, orange indicates females, and blue indicates males. DALY = disability-adjusted life year, ES = exponential smoothing.

### 3.6. Study on the relationship between high BMI and low back pain based on Mendelian randomization

Figures 7 and 8 present the MR analyses based on 473 instrumental SNPs. IVW showed a positive association between genetically predicted BMI and LBP (odds ratio [OR] 1.007, 95% CI: 1.005–1.009; *P* = 6.40 × 10^−13^). The weighted median estimate was similar (OR 1.006, 95% CI: 1.004–1.009; *P* = 1.11 × 10^−5^). MR-Egger and weighted mode estimates were not statistically significant (*P* = .563 and *P* = .111, respectively), whereas the simple mode estimate reached nominal significance (*P* = .044). The consistency of the IVW and weighted median estimates supports the direction of association, although the small effect size and differing assumptions of the sensitivity estimators warrant cautious interpretation.

**Figure 7.**
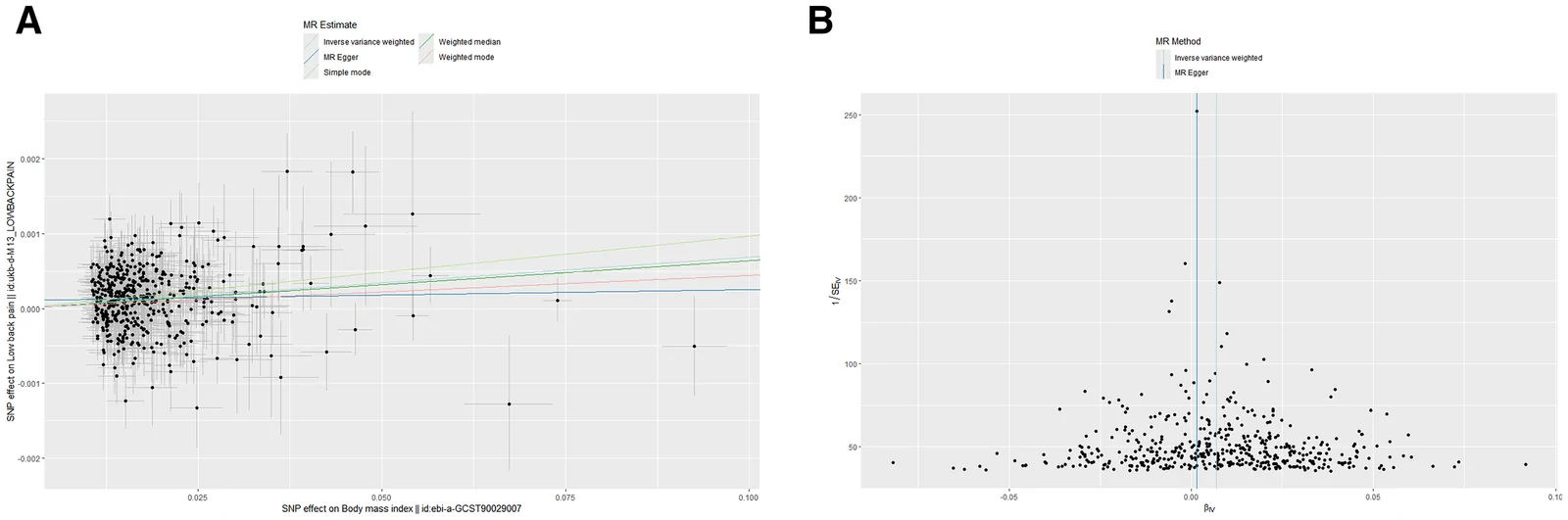
Mendelian randomization analysis of the effect of genetically predicted body mass index on low back pain. Panel A shows the SNP-specific associations and fitted estimates from the MR methods; panel B shows the funnel plot used to assess asymmetry. BMI = body mass index, LBP = low back pain, MR = Mendelian randomization, SNP = single-nucleotide polymorphism.

**Figure 8.**
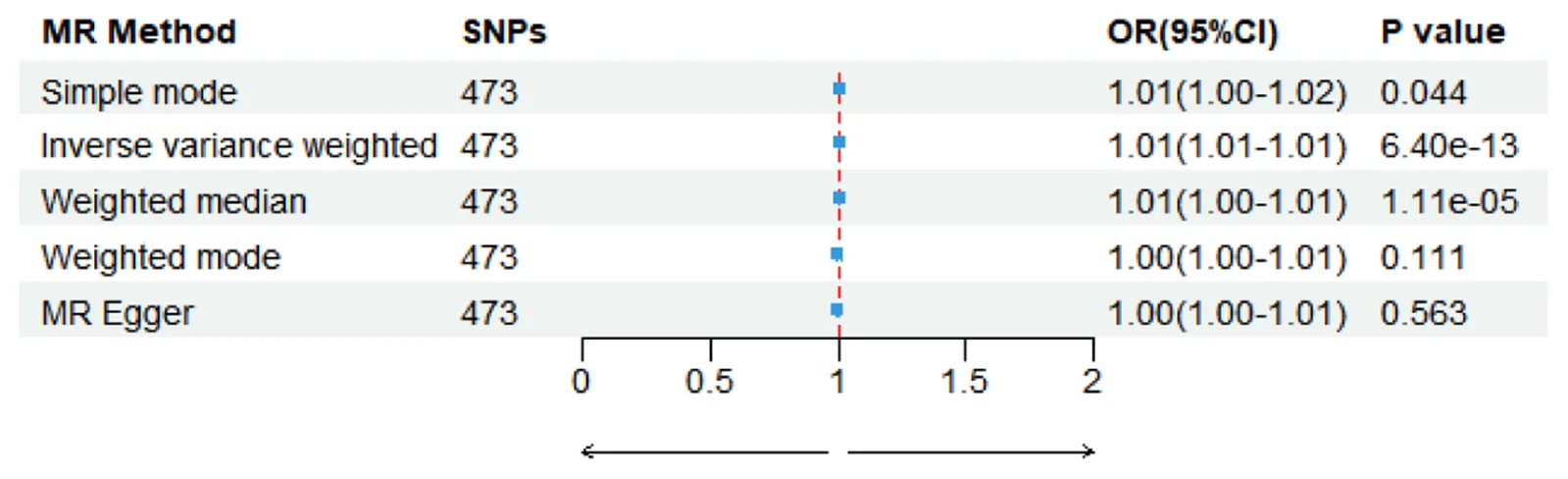
Forest plot of Mendelian randomization estimates for the effect of genetically predicted body mass index on low back pain. CI = confidence interval, MR = Mendelian randomization, OR = odds ratio.

### 3.7. Global consumption of food and beverages in the GDD database

Figures 9–15 summarize the descriptive GDD analyses. SSB intake was highest in the Americas (Fig. 9), whereas refined-grain intake was high in East Asia and North Africa (Fig. 10). Total cereal intake and the relative contribution of grain categories are shown in Figures 11 and 12. Whole-grain intake remained lower than refined-grain intake in many settings (Fig. 13). SSB intake peaked in adolescence and early adulthood and declined at older ages (Fig. 14), with sex-specific patterns shown in Figure 15. These results describe regional dietary context; they do not demonstrate an individual-level diet-BMI-LBP pathway.

**Figure 9.**
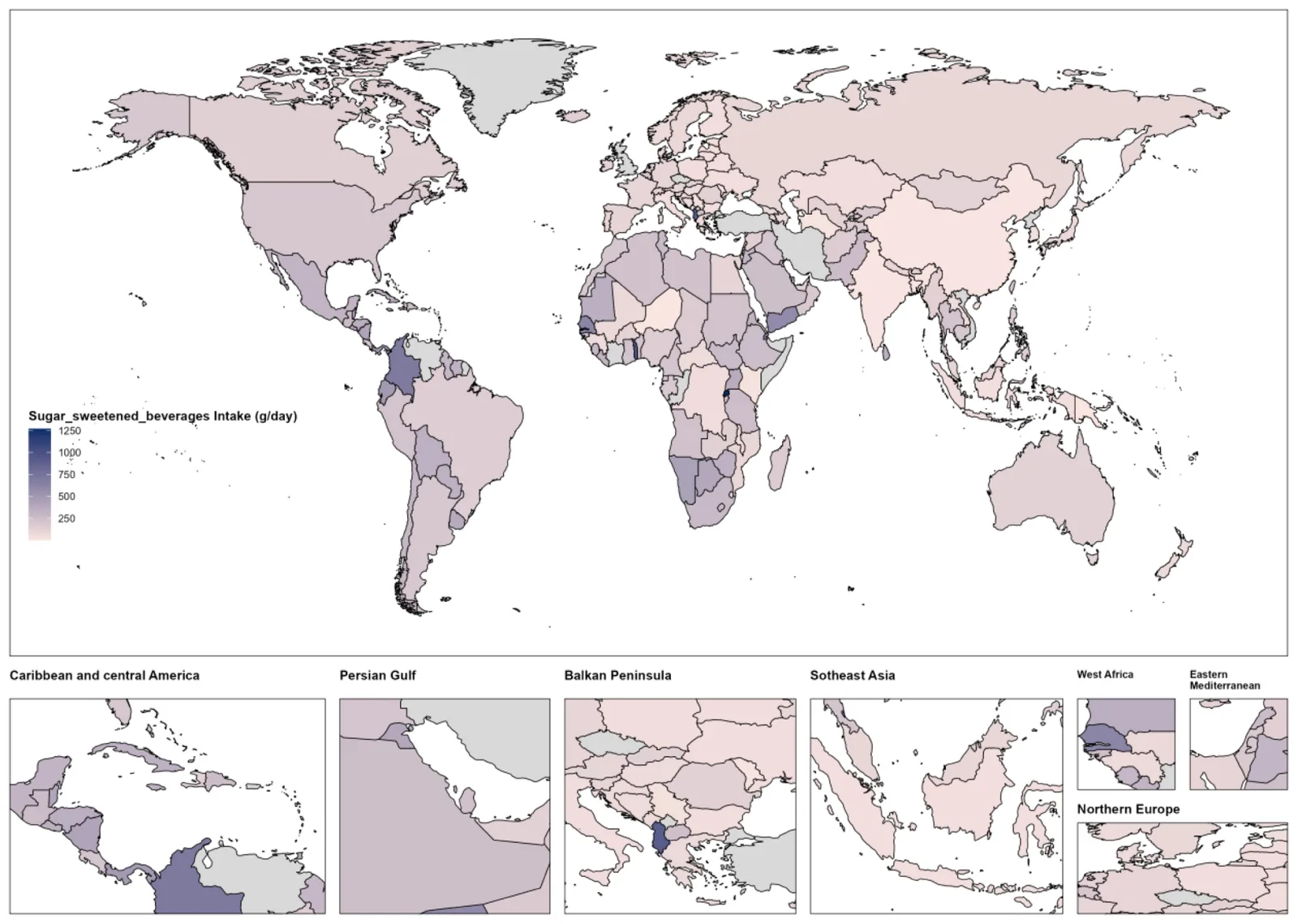
Global distribution of sugar-sweetened beverage intake in the Global Dietary Database. SSB = sugar-sweetened beverage.

**Figure 10.**
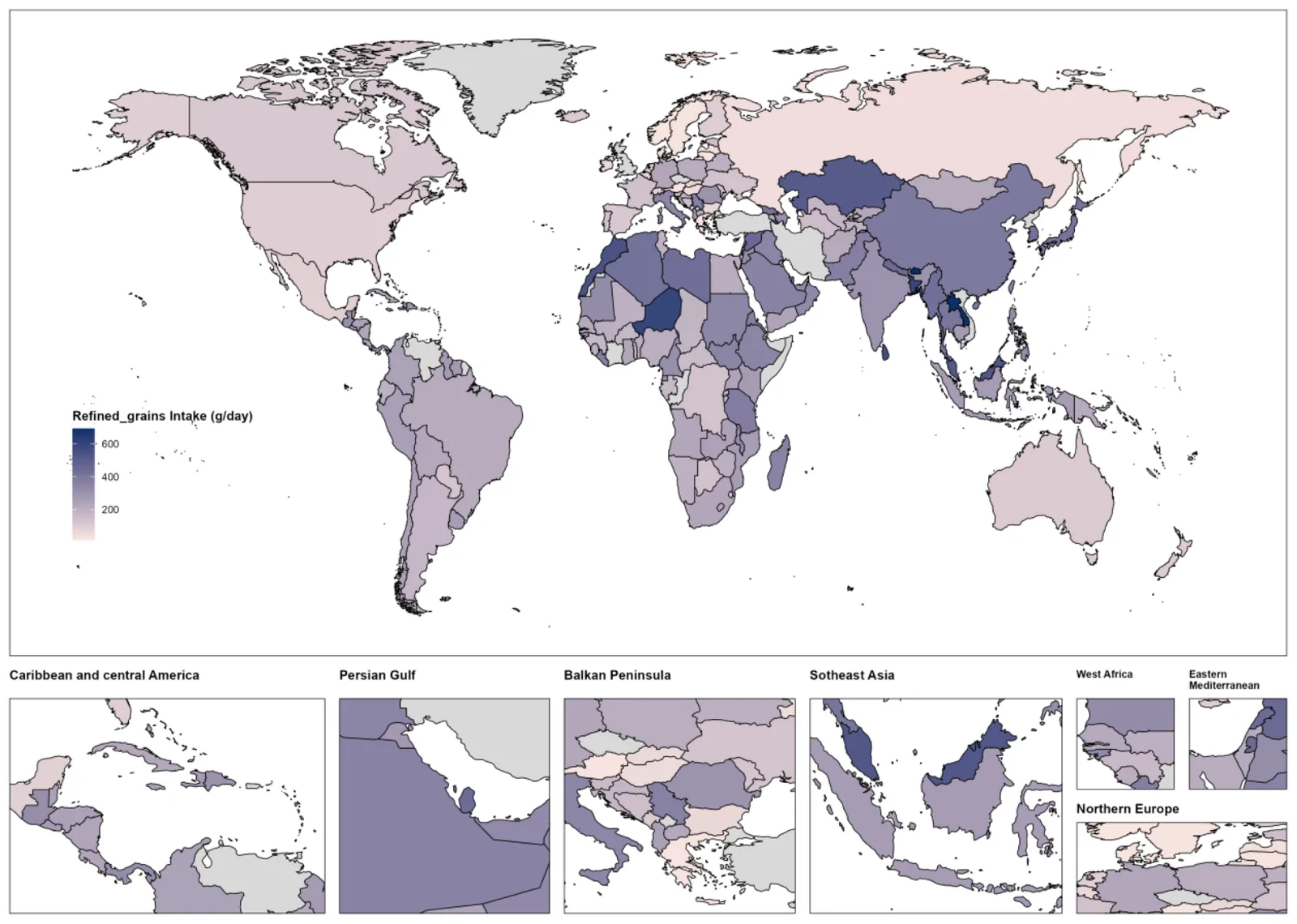
Global distribution of refined-grain intake in the Global Dietary Database.

**Figure 11.**
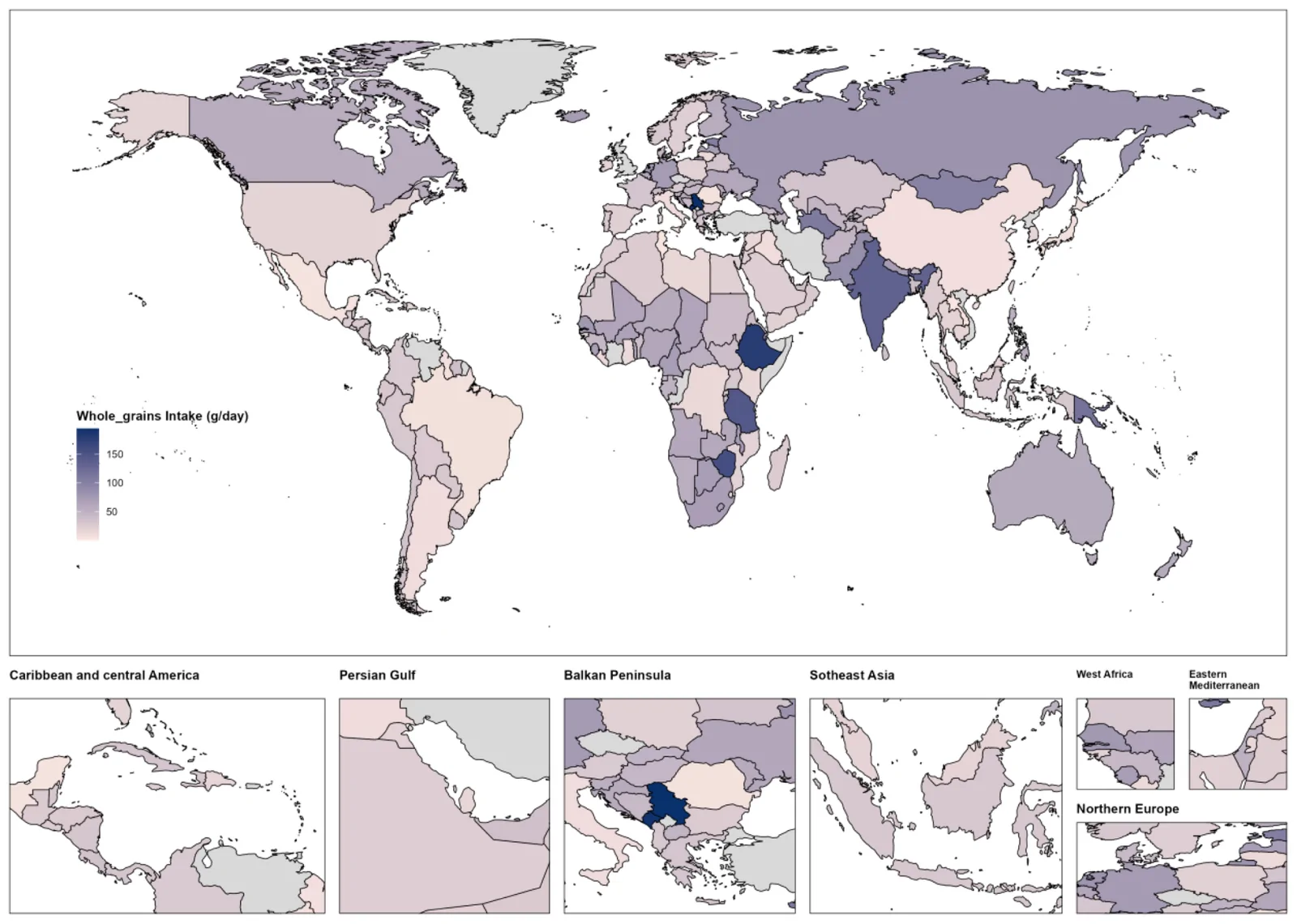
Global distribution of total cereal intake in the Global Dietary Database.

**Figure 12.**
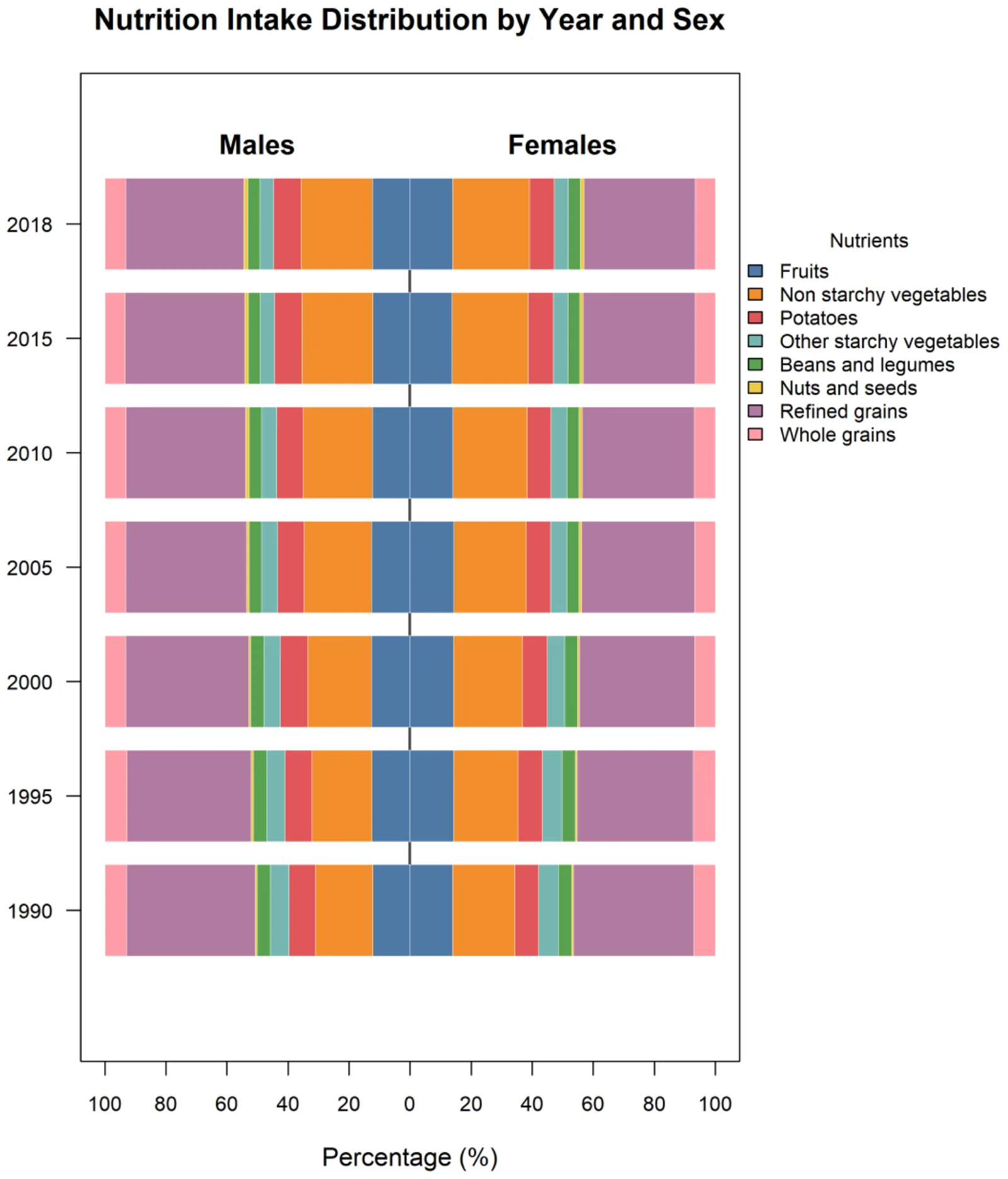
Global composition of cereal intake by grain category and sex in the Global Dietary Database.

**Figure 13.**
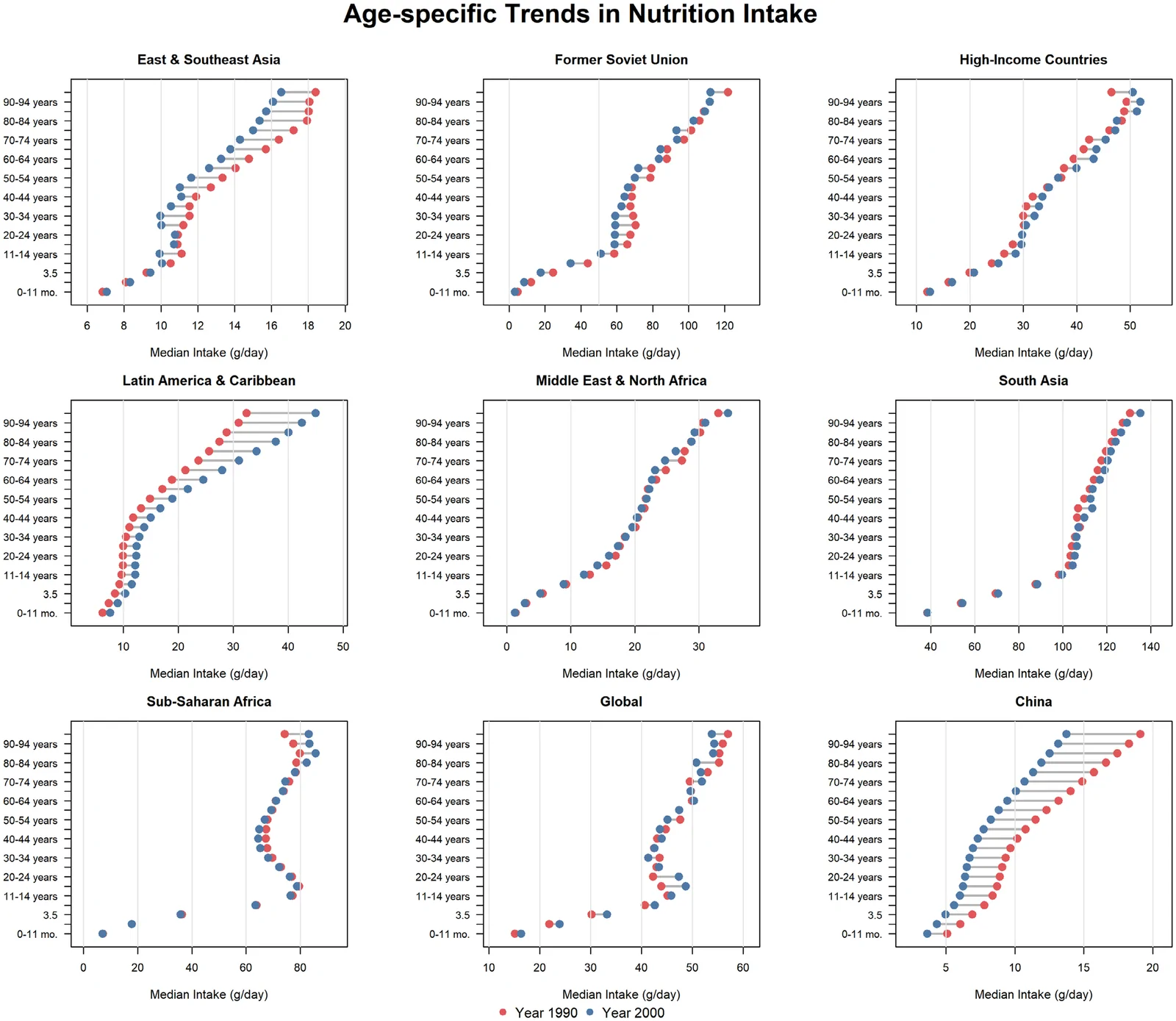
Age-specific whole-grain intake across selected locations in the Global Dietary Database.

**Figure 14.**
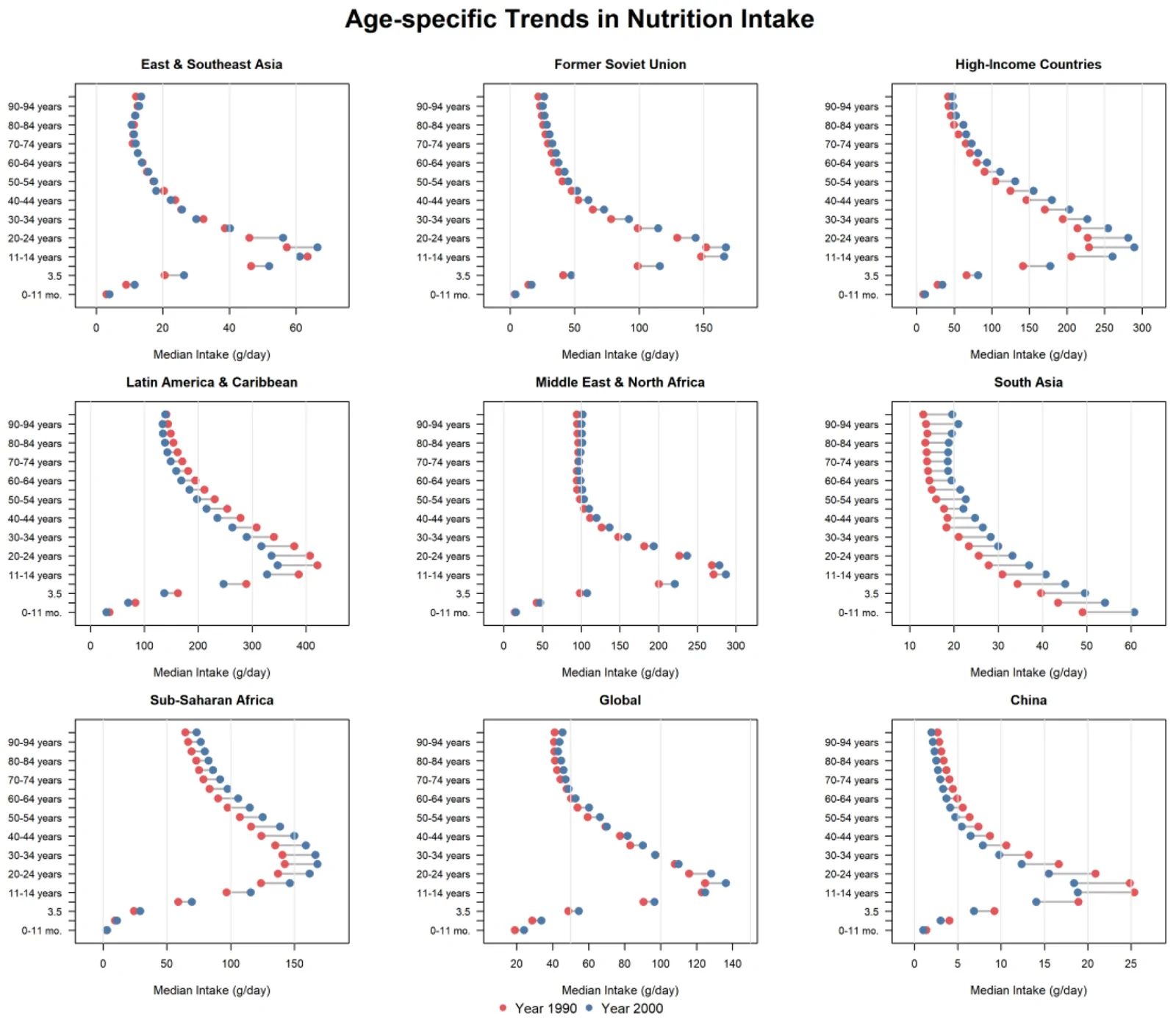
Age-specific sugar-sweetened beverage intake across selected locations in the Global Dietary Database. SSB = sugar-sweetened beverage.

**Figure 15.**
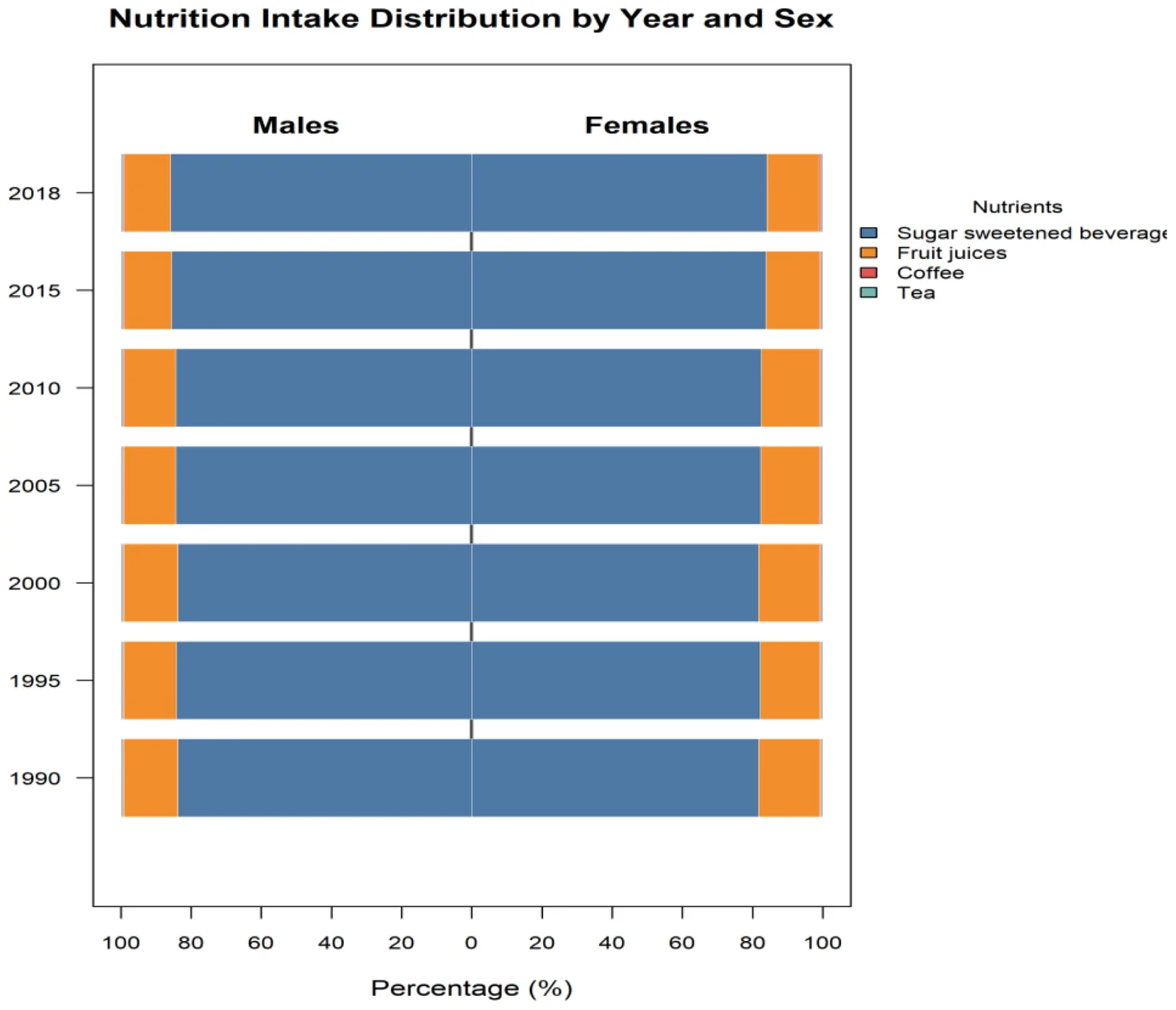
Sex-specific sugar-sweetened beverage intake across selected locations in the Global Dietary Database. SSB = sugar-sweetened beverage.

## 4. Discussion

This study integrated 3 complementary sources of evidence: GBD 2021 estimates of the population burden of LBP attributable to high BMI, GDD estimates of regional dietary patterns, and MR estimates of the effect of genetically predicted BMI on LBP. The burden increased globally and in every examined geographic grouping, with a particularly rapid increase in China. The 3 components answer different questions: GBD quantifies attributable burden, GDD provides ecological context, and MR strengthens causal inference for BMI itself. Their results should therefore be considered together without treating descriptive dietary patterns as proof of mediation.

### 4.1. Necessity and novelty of a multi-database integrative approach

The multi-source design broadens interpretation beyond a single database. GBD 2021 provides internally comparable estimates for 1990 to 2021, while ES offers an explicitly exploratory extension of observed trends. MR reduces, but does not eliminate, confounding and reverse causation when evaluating BMI as an exposure. GDD data add policy-relevant information on dietary patterns that may contribute to high BMI. This structure supports a more cautious evidence chain: population burden, plausible contextual drivers, and genetic evidence for BMI, rather than a single causal pathway from specific foods to LBP.

### 4.2. Key obesogenic dietary drivers and regional heterogeneity

We focused on refined grains and SSBs because they represent distinct, policy-relevant sources of dietary energy. Refined grains are prominent in several East Asian diets, whereas SSB consumption is high in parts of the Americas. The GDD results support region-specific prevention priorities, but the aggregate design cannot determine whether individuals with higher intake subsequently developed high BMI or LBP. The dietary findings should therefore guide hypothesis generation and public health context rather than individual causal attribution.

### 4.3. Biomechanical–metabolic dual-pathway model

Several mechanisms may link higher BMI with LBP. Mechanically, greater body mass increases axial loading and may alter lumbar posture and shear forces.^[13]^ Metabolically, adipose tissue contributes to chronic low-grade inflammation through adipokines and cytokine signaling.^[14,15]^ Experimental and clinical evidence also links inflammatory mediators, matrix-degrading enzymes, and advanced glycation end products with disc degeneration and pain sensitization.^[16-19]^ These mechanisms are biologically plausible and consistent with the MR direction of effect, but they were not measured directly in the present study.^[20,21]^

### 4.4. Biological and biopsychosocial origins of sex differences

The persistent female predominance in absolute burden likely reflects a combination of biological, occupational, and psychosocial factors. Hormonal changes after menopause, differences in body-fat distribution, and sex-specific patterns of musculoskeletal degeneration may contribute.^[22-24]^ Occupational exposures, care responsibilities, anxiety, depression, and differences in pain reporting may also affect observed burden. Importantly, the EAPC comparison showed a steeper global trend in males but no statistically significant male-female difference in China; the higher female burden should not be conflated with a faster rate of increase.

### 4.5. Implications for targeted prevention: dietary and policy strategies

The findings support weight management as 1 component of LBP prevention. In East Asia, increasing substitution of whole grains for refined grains may improve overall diet quality, while in regions with high SSB intake, taxation, marketing restrictions, and access to unsweetened alternatives remain relevant options.^[25,26]^ These measures are justified primarily by their established metabolic benefits; the present ecological dietary analysis does not quantify their direct effect on LBP. Clinical strategies should also combine weight management with physical activity, ergonomic measures, and evidence-based care for chronic pain.

### 4.6. Limitations and future directions

This study has several limitations. First, GBD estimates are model-derived and are less certain in locations with sparse primary data. Second, DALY and YLD estimates were identical for this nonfatal cause, so they should not be interpreted as independent outcomes. Third, the pooled SDI and GDD analyses are ecological; repeated location-year observations, population size, and unmeasured regional factors limit causal interpretation. Fourth, MR estimates reflect lifelong genetic liability and were based mainly on European-ancestry data, which may limit generalizability and do not directly estimate the effect of short-term weight loss. Fifth, the ES projections are extrapolations that do not include policy changes, migration, or other external shocks. Finally, the analytic dataset was frozen using the GBD 2021 cycle. GBD 2023 is now available and may revise historical estimates; independent replication with that cycle is warranted rather than combining estimates from 2 cycles within 1 analysis.

## 5. Conclusion

High BMI contributes to the growing burden of LBP, but the dietary analyses are ecological and should be interpreted as context rather than evidence of mediation. Weight management and region-specific dietary measures may help reduce this burden.

## Author contributions

**Conceptualization:** Dandan Li.

**Data curation:** Dandan Li.

**Formal analysis:** Dandan Li.

**Funding acquisition:** Dandan Li.

**Investigation:** Dandan Li.

**Methodology:** Dandan Li.

**Project administration:** Dandan Li.

**Resources:** Dandan Li.

**Software:** Dandan Li.

**Supervision:** Houzhen Zhang, Dandan Li.

**Validation:** Houzhen Zhang, Dandan Li.

**Visualization:** Houzhen Zhang.

**Writing – original draft:** Houzhen Zhang.

**Writing – review & editing:** Houzhen Zhang.
